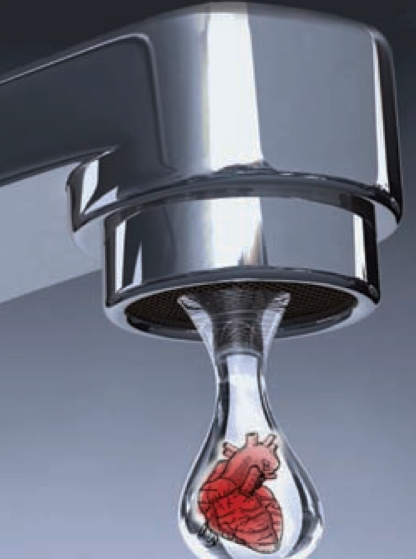# Cardiovascular Health: Hard Data for Hard Water

**DOI:** 10.1289/ehp.116-a114a

**Published:** 2008-03

**Authors:** Adrian Burton

Are people who drink “hard” water containing higher levels of calcium and/or magnesium less likely to suffer cardiovascular disease? This is the question that delegates who attended a World Health Organization (WHO) meeting 21–22 January 2008 in Geneva, Switzerland, are now trying to answer once and for all.

The idea that hard water—particularly that with higher magnesium concentrations—helps ward off cardiovascular problems has been around for 50 years. However, due to the ecologic nature of most studies, uncontrolled confounding factors, and the different variables and outcomes measured, no firm conclusions have ever been drawn. The WHO is therefore coordinating worldwide efforts to compare cardiovascular morbidity before and after changes in the calcium/magnesium content of water supplies.

The aim of the Geneva meeting was to discuss how such a study—ultimately a composite of many smaller studies from different nations—should be performed. “A prospective, multi-country study following a single protocol would be the best way to ensure a sufficiently large sample for overall analysis . . . if we are to make meaningful comparisons,” says Paul Hunter, a professor of health protection at the University of East Anglia, United Kingdom, whose group has been testing a possible protocol.

Hunter’s work involved obtaining mortality and residence data on individuals in areas where notable changes in water hardness had occurred through the introduction or cessation of softening practices, allowing trends in cardiovascular mortality before and after the change in water hardness to be detected.

Controlling the confounding factors in a final meta-analysis involving populations from different countries could pose problems, but “the ‘before and after’ nature of the individual studies should certainly provide meaningful results at the population level,” he says.

The mechanism by which hard water may provide protection against cardiovascular disease remains a matter of debate. The extra calcium it carries could help reduce blood pressure, whereas low serum magnesium concentrations—common to people living in soft-water areas—appear linked with arrhythmias. “Couple this with the fact that many of today’s refined foods are low in magnesium, that many people in developed countries either do not cover or only barely cover their magnesium needs, and that magnesium in drinking water is more bioavailable than that in food, and you can see how [even the relatively small] extra supply of this mineral to people in hard-water areas could be beneficial,” says Frantisek Kozísek, head of the National Reference Centre for Drinking Water in Prague, Czech Republic. “Cooking food in soft water also tends to remove magnesium, calcium, and other essential elements from food, making matters worse.”

The results could lead to countries adopting legislation to supplement drinking water supplies in soft-water areas with calcium and magnesium. Kozísek has already proposed that levels of calcium and magnesium in drinking water be set at 40–80 mg/L and 20–30 mg/L, respectively. “The available evidence suggests these ranges could be beneficial, and . . . there is no evidence that harder water causes any harm,” he explains.

Regu Regunathan, a consultant for the Water Quality Association, says that any recommendations on magnesium or other minerals must be based on absolutely solid data; otherwise, desalination plants and industries providing water softeners and reverse osmosis devices could be needlessly affected. Indeed, soft water has palpable technical advantages over hard water, including reduced scaling in appliances, pipes, and on surfaces, as well as better soap lathering. To this, Kozísek responds, “If health and technical aspects of water are in contradiction, then cost–benefit analyses of the consequences of both aspects should be made to decide what is more important for society.”

## Figures and Tables

**Figure:**